# Lithium Adsorption Using Graphene Oxide: Modeling, Regeneration, and Mechanistic Insights

**DOI:** 10.3390/ma18143211

**Published:** 2025-07-08

**Authors:** Abdulrahman Abu-Nada, Ahmed Abdala, Gordon McKay, Shifa Zuhara

**Affiliations:** 1 Division of Sustainable Development, College of Science and Engineering, Hamad Bin Khalifa University, Doha, Qatar; 2 College of Science and Engineering, Hamad Bin Khalifa University, Doha, Qatar

**Keywords:** sorption equilibrium, kinetic processes, graphene oxide, regeneration, lithium removal

## Abstract

Graphene oxide (GO) was synthesized using the Hummers method and evaluated for lithium-ion removal from aqueous solutions. Characterization via X-ray photoelectron spectroscopy (XPS), Fourier transform infrared (FTIR) spectroscopy, field-emission scanning electron microscopy (FE-SEM), and X-ray diffraction (XRD) confirmed the presence of oxygen-containing functional groups (C–O–C, C=O), which act as active adsorption sites. BET analysis revealed a surface area of 232 m^2^/g and a pore volume of 0.4 cm^3^/g, indicating its high porosity. Lithium adsorption was tested using synthetic Li-doped solutions under controlled conditions. Kinetics and equilibrium studies demonstrated that the process followed the pseudo-second-order model and the Redlich–Peterson isotherm, achieving an optimum lithium adsorption capacity of 179 mg/g. The adsorption efficiency was influenced by factors such as pH and salinity. Regeneration experiments showed that HNO_3_ was the most effective desorbing agent, enabling GO to be reused multiple times with a moderate loss of adsorption capacity. These findings highlight GO’s exceptional efficiency in lithium removal and its suitability for wastewater treatment applications. Its recyclability and reusability further support a circular economy, making GO a highly promising material for sustainable lithium recovery and broader environmental remediation efforts.

## 1. Introduction

With the increasing global use of electric vehicles and other applications, the demand for lithium is projected to rise significantly; its value is also anticipated to increase substantially [1]. Lithium has applications in medicine, where it is used in the treatment of diseases such as Alzheimer’s and bipolar disorder [2]. It is also most commonly known for its recent applications in batteries and microelectronics [3]; other known uses include alloys, ceramics, air conditioning, and weapons [4].

Lithium reserves have been significantly depleted due to their uneven distribution within the Earth’s crust [5]. In 2015, lithium resources were estimated to exceed 47 million tons, with Argentina, Bolivia, and Chile serving as the primary sources [6]. Alternative lithium resources include produced water from the gas and oil exploration industries, brine, lithium ore deposits, and seawater [1,7]. The removal and recovery of lithium from wastewater has been explored through the use of membranes, molecular sieve-based processes, and adsorption technologies, employing a range of materials in these processes. These methods have been developed with an emphasis on increasing selectivity to recover lithium ions, given the challenges posed by the presence of electrolytes that complicate the separation process. However, the success rates of these technologies have varied.

Shaped titanium-based lithium-ion sieves (HTO-P) were evaluated for lithium ion (Li^+^) recovery from the brine of Qinghai West Taijinar Salt Lake, achieving a lithium adsorption capacity of 19.0 mg/g at 30 °C [8]. Layered lithium–aluminum hydroxides combined with an ion exchange resin were evaluated using simulated water, demonstrating an adsorption capacity of 2.0 mg/g [9]. The adsorbent was also evaluated using old brine from Qarhan Salt Lake in a separate study, where it demonstrated an uptake capacity of 7.27 mg/g and exhibited highly effective selectivity for lithium over calcium, potassium, sodium, and magnesium. SiO_2_ ion-imprinted polymer demonstrated a higher maximum capacity of 4.1 mg/g [10]. Spherical layer-structured H_2_TiO_3_ ion sieve (LSTIS) particles were tested on a solution containing multiple cations and exhibited a more preferential selectivity for Li^+^ over Ca^2+^, K^+^, Mg^2+^, and Na^+^, with a lithium adsorption capacity of up to 30.0 mg/g [11]. Eco-friendly chitosan (CTS) has been studied for the preparation of a granulated H_4_Mn_5_O_12_ (HMO) ion sieve, which was investigated using geothermal water containing various cations. The lithium adsorption capacity was reported to be 12.0 mg/g [12]. A synthetic ion exchange resin was evaluated using a laboratory-scale fixed bed column arrangement for lithium recovery, achieving a lithium adsorption capacity of 3.0 mg/g [13]. Recyclable magnetic Fe-doped manganese oxide lithium ion-containing sieves with a spinel structure have been tested for lithium removal in aqueous solutions, achieving a maximum capacity of 35.0 mg/g [14].

Chemically treated activated carbon (CAC), enhanced by the addition of manganese dioxide (MnO_2_) nanoparticles, has been prepared using varying concentrations of manganese salts. The material exhibited improved performance with higher Mn loadings, achieving a capacity of 88.5 mg/g [15]. Lithium adsorption onto manganese oxide has been studied using seawater, achieving a capacity of 11.0 mg/g despite low lithium concentrations and the presence of high levels of competitive cations [16]. Date pits impregnated with cellulose nanocrystals and ionic liquid demonstrated a lithium uptake capacity of 99 mg/g, making it the most effective adsorbent for lithium removal to date [17].

Membrane-based technologies face several limitations, including high operational expenses (OPEX) caused by membrane fouling, leakage of organic solvents due to the use of chemical reagents in the desorption process, low adsorption capacities, and leakage of inorganic particles. Additionally, operating costs tend to increase with higher salinity, which is common in brines, and desorption efficiencies are often low [18]. This study introduces the novel use of produced graphene oxide (GO) as a potential adsorbent for lithium removal from aqueous solutions. By far, the majority of studies on lithium adsorption on graphene and graphene oxide are based on Li-ion batteries and the production of Li-graphene electrodes, utilizing theoretical studies, including molecular simulations, Density Functional Theory, and Monte Carlo Simulations [19,20,21]. The application of graphene oxide to remove lithium from effluent wastewater by adsorption has received limited attention, with the potential to recover lithium through a regeneration process. For example, a highly complex adsorbent was developed and tested using sulfonated graphene oxide and Ti_3_C_2_T_x_ MXene nanocomposite hydrogels to adsorb lithium from wastewater [22], but the uptake capacity of 46 mg/g was only reasonable. The adsorption of lithium ions onto GO has been examined using various characterization techniques to evaluate the adsorbent’s properties. These tests provided insights into the crystalline nature of the graphene oxide structure, the BET surface area, the material’s chemistry, and the availability and extent of functional groups on GO. The GO product has been investigated in a series of experiments using different process parameters to assess its lithium adsorption capability. The experimental results have been analyzed using kinetic and isotherm models to determine which model is most appropriate for describing the various exchange and sorption mechanisms.

## 2. Materials and Methods

### 2.1. Materials

Lithium solution (Sigma Aldrich, St. Louis, MO, USA) and nitric acid (HNO_3_, 15.7 M) (Sigma Aldrich, St. Louis, MO, USA) were utilized for the preparation of lithium standards. A standard solution, 1.0 g/L Li^+^, was made from lithium chloride (LiCl) (Sigma Aldrich, St. Louis, MO, USA) by dissolving in deionized water. Natural flake graphite (XFNano, Nanjing City, China, 99% purity), sulfuric acid (Fisher Chemical, Hampton, NH, USA, 95%), potassium permanganate (KMnO_4_) (Sigma Aldrich, St. Louis, MO, USA), sodium nitrate (NaNO_3_) (Sigma Aldrich, St. Louis, MO, USA), and hydrogen peroxide (H_2_O_2_) (Sigma Aldrich, St. Louis, MO, USA, 30%) were employed in the preparation of graphite oxide. Hydrochloric acid (HCl) (Honeywell 37%) and sodium hydroxide solution (NaOH) (Sigma Aldrich, 50%) were also used in the process.

Hummer’s method [23] was used to synthesize the GO [19]. In brief, 1 g of graphite and 1 g of sodium nitrate (NaNO_3_) were combined with 40 mL of sulfuric acid (H_2_SO_4_). Next, 6 g potassium permanganate (KMnO_4_) was slowly added to the reactor at a constant temperature of 35 °C and stirred for 1 h. Afterward, 80 mL of milli-Q water was added, and the solution was stirred for 30 min at 90 °C. Subsequently, 6 mL of hydrogen peroxide (H_2_O_2_) and 150 mL of Milli-Q water were slowly introduced. The resulting mixture was then washed thoroughly with deionized water, followed by several cycles of centrifugation at 10,000 rpm and sonication.

The active surface groups on the GO were characterized by X-ray photoelectron spectroscopy (XPS) (Thermo Fisher, ESCALAB, 250Xi, Waltham, MA, USA) and Fourier-transform infrared (FTIR) spectroscopy (Thermo Scientific Nicolet iS50, Waltham, MA, USA). The crystallinity of GO was analyzed through powder X-ray diffraction (XRD) (Rigaku SmartLab, Akishima, Tokyo, Japan). Surface area, porosity, and pore size distribution were measured via the BET method (Micromeritics ASAP 2020 Plus, Atlanta, GA, USA). Thermogravimetric analysis (TGA) (Discovery SDT-650, New Castle, DE, USA) was used to evaluate thermal stability, and scanning electron microscopy (SEM) (JEOL 7610F, Akishima, Tokyo, Japan) was employed to study the GO morphology.

### 2.2. Adsorption Measurement

A standard solution of 1.0 g/L Li^+^ was produced from lithium chloride (LiCl) salt dissolution in DI water. From this, 50 mL samples were prepared with initial lithium concentrations ranging from 40 to 220 mg/L. A specified dose of graphene oxide (GO) was added to each sample, which was then agitated at 500 rpm for 120 min at 25 °C using a magnetic stirrer. The lithium concentration in the samples was measured using ICP-OES (Agilent Technologies 5110). Before measurement, the samples were centrifuged for 5 min, filtered through a 0.22 µm filter, and diluted to concentrations below 10 ppm. The lithium concentration was obtained from a calibration line plotted from Li^+^ standard samples ranging from 0 to 10 ppm.

The kinetics experiments were conducted by agitating 100 mL of lithium chloride solution at concentrations from 40 mg/L to 220 mg/L, using a fixed dose of GO. The samples were taken at selected time intervals to measure the Li^+^ concentrations. To determine the optimal pH for adsorption, a series of experiments was performed in the pH range of 3 to 11, with pH adjustments made by adding 0.1 M HCl and 0.1 M NaOH. The impact of salinity on the adsorption process was evaluated at sodium ion (Na^+^) concentrations of 1000 mg/L and 5000 mg/L, prepared using analytical-grade sodium chloride (NaCl).

For recyclability assessment, the GO adsorbent was regenerated over three cycles using 1 M solutions of HCl and HNO_3_.

The adsorption uptake capacity (q_t_) at a defined time t and the percentage adsorbed were evaluated using the formulas in Equations (1) and (2):

(1) $$q_{t}=\frac{(C_{0}-C_{t})V}{W}$$

(2) $$Removal\;\%=\frac{\left(C_{0}-C_{t}\right)}{C_{0}}\times 100\;$$

where *C*_0_ (mg/L) and *C_t_* (mg/L) represent the initial and final lithium solute concentrations, respectively, *V* (mL) is the solution volume, and *W* (mg) is the mass of adsorbent. All experimental results are the average of triplicate measurements with a margin of error of ±5%.

## 3. Results and Discussion

This section can be divided into a precise description of the experimental results, their interpretation, as well as a discussion of the results and the application of kinetic modeling and equilibrium isotherm modeling.

### 3.1. GO Characterization

**Figure 1:**
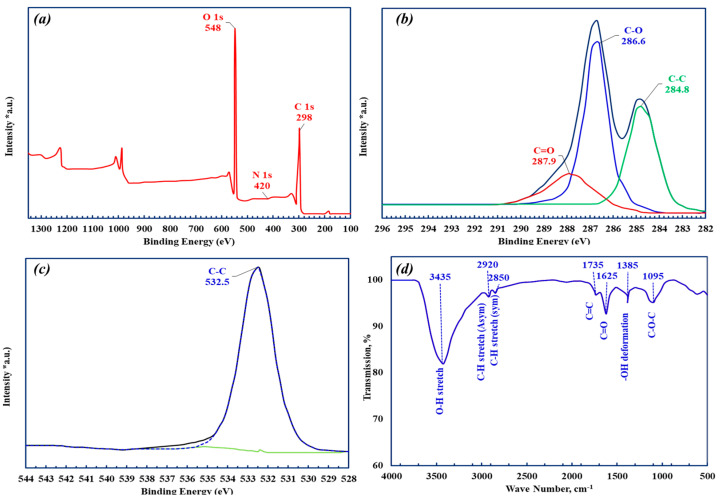
XPS (**a**) survey spectrum and higher resolution (**b**) C 1s and (**c**) O 1s spectra, and (**d**) FTIR spectrum of GO, reproduced from [24].

In this study, we used a commercial GO sample that was fully characterized in our previous work [24]. For completeness, a summary of the characterization is provided in this section. The XPS survey spectrum, presented in Figure 1a, confirms the composition of the elements in the GO, with oxygen concentration ranging from 37% and carbon content of 63%. The C/O ratio of 1.7 supports the efficient oxidation of graphite into GO, but is slightly lower than the typical 2/1 ratio. The core spectra were obtained using a pass energy of 20 eV and a step size of 0.1 eV. The survey spectrum, covering a range from 5 eV to 1350 eV, revealed the presence of signals from C, Mn, and O. The manganese signal was attributed to residual potassium permanganate, which was used as an oxidizing agent during the GO synthesis. The XPS deconvoluted C1s spectrum, Figure 1b, displayed distinct peaks for GO, including a C–C peak at 284.7 eV, a prominent C–O peak at 286.6 eV, and a weaker C=O peak at 287.8 eV. Additionally, the deconvoluted O 1s spectrum (Figure 1c) confirms the presence of carbonyl oxygen groups.

The FTIR scan spectrum of GO, presented in Figure 1d, aligns with the consistently reported spectrum of GO. The peak at 1725 cm^−1^ is associated with C=O carbonyl/carboxyl groups, while the peak at 1093 cm^−1^ can be assigned to the C–O–C epoxide groups. The 1632 cm^−1^ peak corresponds to aromatic C=C bonds, and the broader peak band at 3436 cm^−1^ is attributed to the hydroxyl (-OH) groups. These FTIR findings provide additional confirmation of the successful oxidation of graphite to GO (Table 1).

**Figure 2:**
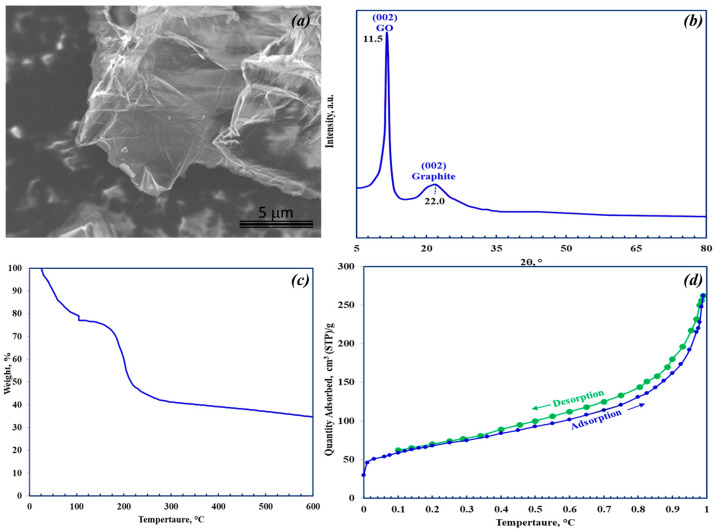
Characterization of GO used in the study: (**a**) XRD X-ray diffraction pattern; (**b**) FTIR analysis; (**c**) TGA thermal stability curve; and (**d**) BET nitrogen adsorption/desorption isotherms for GO at 77K, reproduced from [24].

The GO morphology was investigated by SEM analysis. As shown in the SEM Figure 2a, GO consists of large, irregular shapes resembling flakes or sheet-like structures with wrinkled surfaces. Sheets with dimensions ranging from 500 nm to several µm are visible. It is worth noting that the SEM images in Figure 1b were obtained from a dry powder form of the synthesized graphite oxide, rather than the dispersion of the exfoliated GO form. In this dry powder form, the graphite oxide tends to appear as large, aggregated clusters of flakes, which obscures the visualization of individual sheet structures in SEM micrographs.

XRD analysis was performed to investigate the crystalline structure of GO. The GO XRD diffraction pattern (Figure 2b) exhibits a prominent diffraction peak at 2θ ≈ 11.5° associated with the diffraction from the graphite oxide (002) plane, indicating an expanded interlayer spacing from 3.34 Å for graphite to 7.7 Å due to the presence of the oxygenated functional groups and the intercalation of strongly adsorbed water molecules [24]. Moreover, the crystallite length was 15.8 nm, as determined by Scherrer’s Law. The number of GO layers could be estimated from the ratio of the mean crystallite length to the d-spacing, resulting in approximately 20 layers, which are in reasonable agreement with findings from other GO samples [24,25,26]. It is worth noting that, in addition to the GO sharp (002) peak, a low intensity and broad peak is observed at 2θ ≈ 22° associated with the (002) graphitic peak, suggesting incomplete oxidation and consistent with the C/O ratio of <2.

The thermal stability of GO was examined by thermogravimetric analysis (TGA), and the resulting profile is shown in Figure 2c. This analysis is crucial for assessing the potential for thermally regenerating adsorbed lithium and determining the optimal temperature to degas the sample during the BET study without damaging the GO adsorbent. This study revealed that the GO adsorbent contained approximately 15% moisture. GO demonstrated thermal stability up to 160 °C, beyond which the rate of degradation of the sample increased significantly, reaching a maximum at 216 °C. This was attributed to a decrease in the level of oxygen-containing functional groups. After this temperature, the degradation rate slowed sharply. Similar thermal stability behavior for GO has been observed in other studies [26].

The surface area, pore size, and pore size distribution play crucial roles in the performance of adsorbents [27]. The surface area directly influences the adsorption capacity, while pore size can limit the diffusion of adsorbate molecules depending on their size. The GO sample was analyzed using N_2_ adsorption at 77 K, and its surface area was determined using the Brunauer–Emmett–Teller (BET) method [28]. The pore volume and pore size distribution were assessed using the Barrett–Joyner–Halenda (BJH) method. The isotherm plot in Figure 2d displays a hysteresis loop, which is indicative of mesoporosity, having a surface area of 232.3 m^2^/g. The pore size, 6.79 nm, falls within the mesoporous range. This finding aligns with the characteristics of the N_2_ adsorption and desorption isotherms. Additionally, the specific surface area is relatively high compared to many other carbon-based adsorbents, which enhances the adsorption properties of GO [29].

### 3.2. Adsorption Studies

The use of GO for lithium-contaminated water was explored, and the impacts of several factors, such as the mass of GO, the contact time, salinity, solution pH, and the initial concentration of lithium, were assessed.

#### 3.2.1. Effect of GO Dosage

**Figure 3:**
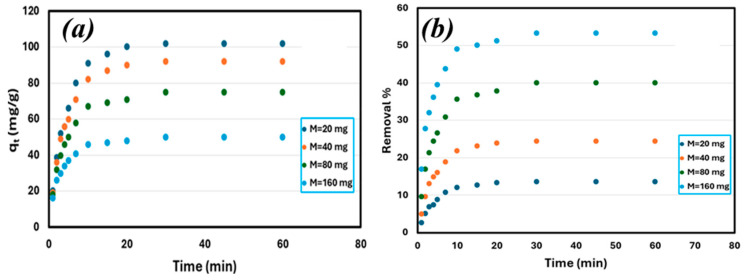
Influence of GO dose on (**a**) Li adsorption capacity and (**b**) efficiency for lithium removal: initial C_0_ = 150 mg/L, volume = 100 mL, magnetic stirrer speed = 500 rpm, T = 25 °C, and initial pH = 5.

The rate and adsorption capacity of Li^+^ were determined, as shown in Figure 3a, using a range of GO doses (20, 40, 80, and 160 mg/100 mL solution volume) with an initial Li^+^ concentration of 150 mg/L. Within 30 min, the systems reached equilibrium, and the specific Li^+^ adsorption capacity increased as the GO dosage decreased.

Conversely, Figure 3b shows the percentage of Li^+^ removed from each sample, and as expected, the greater the mass of GO, the higher the percentage of Li^+^ removal. At an adsorbent concentration of 20 mg/100 mL, the percentage removal is 12%. At a dosage of 160 mg/100 mL, the percentage removal increases to 55%. The removal percentage of 55% is highly effective for such a small adsorbent dose of 1.6 g/L.

#### 3.2.2. Effect of pH

**Figure 4:**
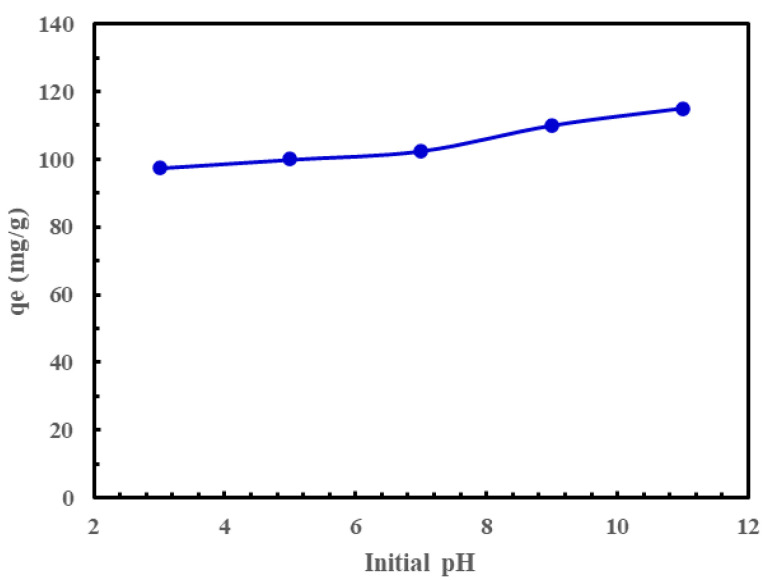
Influence of initial solution pH on the uptake capacity for Li^+^ on GO. Solution volume = 50 mL, T = 250 °C; magnetic stirrer speed = 500 rpm; initial Li^+^ concentration, C_0_ = 200 mg/L; GO mass = 10 mg.

Figure 4 illustrates the impact of initial pH on the adsorption capacity of Li^+^ onto GO. The adsorption experimental studies were conducted at pH levels of 3, 5, 7, 9, and 11, with pH 11 showing the highest adsorption capacity. However, the increase in capacity at pH 11 was not significant. This suggests that the adsorption of lithium on GO is relatively unaffected by the initial pH of the solution.

### 3.3. Kinetic Modeling

Kinetic modeling offers valuable insight into the adsorption mechanisms, including mass transfer and chemical interactions between lithium ions and GO. To explore the kinetic mechanisms governing the adsorption of lithium onto GO, various kinetic models were applied to the experimental data. Each model is based on distinct mechanistic assumptions, as follows: pseudo-first-order (PFO) [30], Avrami [31], pseudo-second-order (PSO) [32], Elovich [33], and the intraparticle diffusion model [34,35] (Table S2).

**Figure 5:**
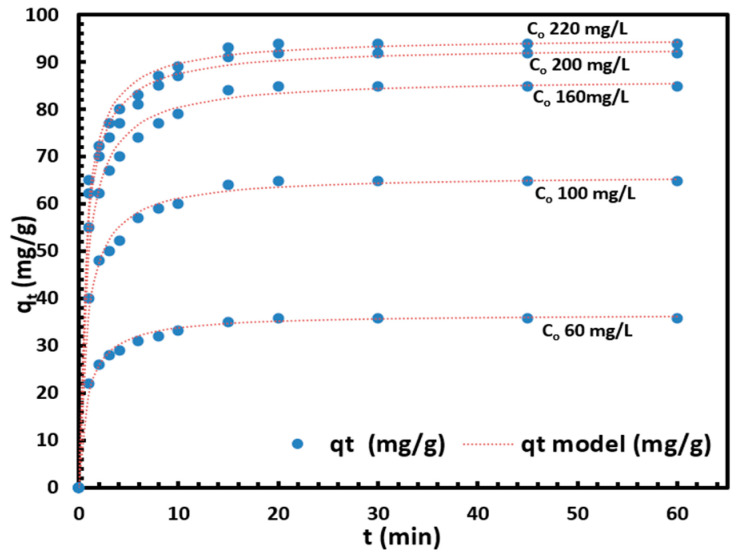
Pseudo-second order kinetic models fitting of adsorption of different initial concentrations of Li^+^ on GO. Solution volume = 50 mL, T = 25 °C; magnetic stirrer speed = 500 rpm; initial Li^+^ solution pH = 5.0; GO mass = 10 mg.

Figure 5 shows the fit of the pseudo-second-order (PSO) model, where the GO was found to match the data exceptionally well across the entire adsorption period, as indicated by the very high correlation coefficient and the lowest SSE. The results for each kinetic model are summarized in Table 2. Non-linear forms of the kinetic models were used for fitting, except for the pseudo-first-order model. The best-fit model was determined based on the sum of squared errors (SSE) and the correlation coefficient (R^2^) values for the applied models. The pseudo-first-order model showed the poorest fit, likely due to its assumption of a single mechanism driving the reaction throughout its duration. The assumptions and mechanisms of these models are discussed in Section 3.8 on lithium adsorption.

The calculated equilibrium capacity for the PSO model is presented in Table 2, and it closely matches the values obtained from the experimental data, further confirming that the PSO model provides the best fit to the Li adsorption process.

The pseudo-second-order kinetic model indicates that the Li adsorption process is driven by the difference between the equilibrium adsorption capacity (q_e_, mg/g) and the adsorption capacity at time t (q_t_, mg/g), with the adsorption rate being proportional to the square of this driving force.

**Figure 6:**
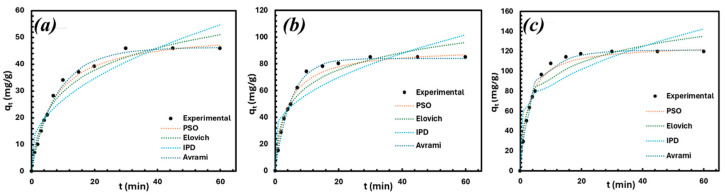
Kinetic models for Li^+^ adsorption on GO for three initial Li^+^ concentrations. Solution volume = 50 mL at 250 °C; magnetic stirrer speed = 500 rpm; initial Li^+^ solution pH = 5.0; GO mass = 10 mg. Initial Li^+^ concentrations of (**a**) = 40 mg/L; (**b**) = 100 mg/L; and (**c**) = 200 mg/L.

Figure 6a–c display the kinetic model plots at low, medium, and high lithium ion concentrations, respectively. These figures demonstrate that different models provide relatively better fits at different concentrations, and the quality of the fits is influenced by the chosen error analysis method. As expected, this variation aligns with the assumptions inherent in each model and the specific error analysis approach used. In Figure 6a, for the initial concentration of 40 mg/L, the model fits are generally good at lower capacity values and around the bend in the isotherm. However, at higher capacity loadings, the fits tend to degrade. For the initial concentration of 100 mg/L in Figure 6b, the fits around the isotherm bend are not as strong for several models, but the fits for high-capacity data show significant improvement. At the high concentration of 200 mg/L, as shown in Figure 6c, the fittings for most models have improved substantially and are now quite accurate. Additionally, comparing the error analysis for the PSO model at different concentrations and capacities shows that the sum of squared errors (SSE) favors high concentrations and capacities, with lower SSE values observed at these higher concentrations.

### 3.4. Adsorption Isotherm

Equilibrium isotherm experiments were conducted with initial Li^+^ concentrations varying from 40 to 200 mg/L, while other conditions, including an initial pH of 5, were maintained constant. The results indicated that higher initial lithium concentrations resulted in higher maximum adsorption capacities. Each experiment used a solution volume of 50 mL and an adsorbent dosage of 10 mg. The solutions were stirred at 500 rpm using a magnetic stirrer at room temperature. Several classical equilibrium equations were analyzed, namely, Langmuir [36], Freundlich [37], Redlich–Peterson [38], Sips or Langmuir–Freundlich [39], and Temkin [40] (Table S1).

**Figure 7:**
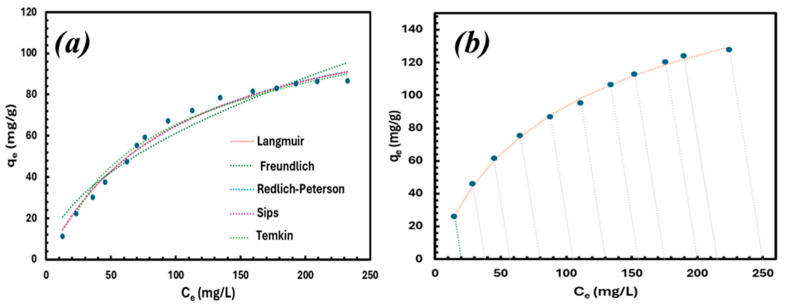
(**a**) Comparison of the isotherms and (**b**) R-P model operating lines (Equation (4)) fitted to the experimental equilibrium results for adsorption of Li^+^ on GO.

Figure 7 shows the different isotherm curves applied to the equilibrium data. Among them, the Freundlich isotherm provided the weakest fit, suggesting that multilayer adsorption is not the primary mechanism. Table 3 presents the equilibrium data fitted to various isotherm models to identify the most accurate explanation for the adsorption process. The Redlich–Peterson isotherm provided the best fit, with the highest correlation coefficient and the lowest sum of squared errors. The Sips model provided a close fit, as it combines aspects of both the Freundlich and Langmuir isotherms. Its comparison with experimental data is depicted.

The Redlich–Peterson (RP) isotherm merges the features of the Freundlich and Langmuir isotherms, deviating from the ideal monolayer adsorption behavior described by the Langmuir model. Instead, it accommodates a broader spectrum of adsorption mechanisms, demonstrating a linear relationship with concentration over a wide range. The non-linear forms of different isotherm models were applied to fit the experimental data. The mathematical expression for the RP isotherm is given by the following:

(3) $$q_{e}=\frac{K_{R}C_{e}}{1+a_{R}C_{e}^{\mathrm{bR}}}$$

where K_R_ (L/g) and a_R_ (L/mg) are constants of the Redlich–Peterson isotherm, and b_R_ is the isotherm exponent. The model tends to behave like the Freundlich isotherm as b_R_ approaches 0 and resembles the Langmuir isotherm as b_R_ approaches 1. The function is represented by an empirical expression, which is characterized by a consistent slope of the operating lines across all experiments, as shown by the following:

(4) $$q_{e_{C01}}=-5\times (C_{01}-C_{e1})$$

In this equation, C_01_ represents the specific initial lithium concentration in mg/L, C_e1_ is the equilibrium concentration corresponding to the given initial concentration in mg/L, and $q_{e_{C01}}$ is the equilibrium capacity at the respective initial concentration in mg/g. Changes in the initial concentration values result in parallel operating lines, each maintaining a constant slope of −V/W = 5, as shown in Figure 7b.

### 3.5. Effect of Salinity

The effect of salinity on lithium adsorption was investigated to simulate real wastewater conditions, where various salts, including sodium chloride (NaCl), are present. Two salinity levels were tested: 1000 ppm and 5000 ppm, using a GO dosage of 20 mg and a solution volume of 100 mL, prepared by dissolving the appropriate amount of salt in deionized water. The initial lithium concentration was set at 200 mg/L. The solutions were stirred at 500 rpm using a magnetic stirrer and maintained at 25 °C, with an initial pH of 5. The experiments were conducted over a period of 2 h.

The results showed a reduction in the maximum adsorption capacity of lithium by 27.1% and 47.9%, yielding values of 87.5 mg/g and 62.5 mg/g at Na^+^ concentrations of 1000 ppm and 5000 ppm, respectively. This decrease in lithium adsorption was likely due to the repulsion between the positively charged Na^+^ ions and the lithium ions in the solution.

### 3.6. Comparison of Adsorption Capacities

Table 4 summarizes the performance of other competitive adsorbents for lithium removal in aqueous solution at different concentrations, listing their uptake sorption capacities and the best-fitting equilibrium correlations. The synthesized GO in this study outperformed these alternatives, offering superior efficiency and an environmentally sustainable solution.

### 3.7. Regeneration Studies

**Figure 8:**
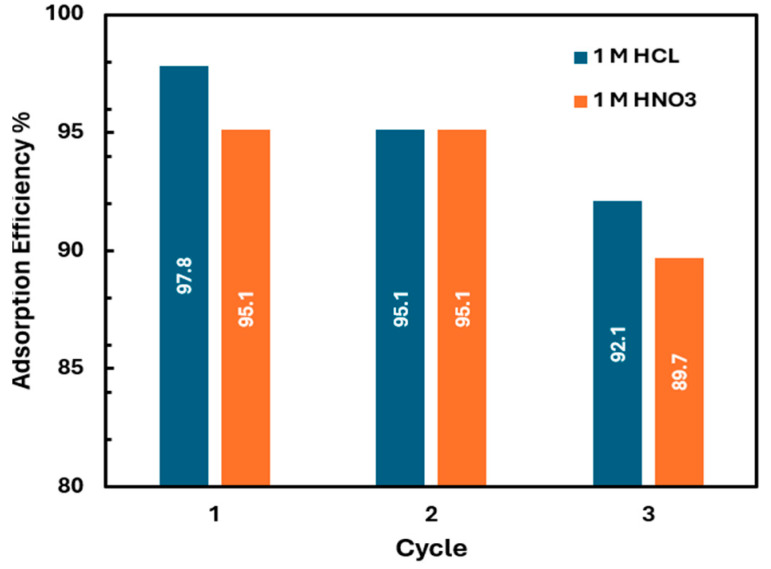
Adsorption efficiencies of GO for Li^+^ removal after 3 regeneration cycles.

To assess the reusability of GO after Li^+^ adsorption, it underwent three adsorption/desorption cycles. Lithium desorption was performed using 1 M HCl and 1 M HNO_3_ as eluents. After each cycle, the GO was filtered and immersed in the respective eluents to evaluate desorption efficiency. Following desorption, the GO was filtered, rinsed three times with deionized water, and then dried at 60 °C. The washing solution was analyzed to determine the ion concentration. The desorption efficiency after three cycles was 88.9% for HCl and 82.8% for HNO_3_. Figure 8 illustrates the decrease in adsorption efficiency after each cycle.

The recycling performance of GO for lithium adsorption was evaluated over three cycles using 1 M HCl and 1 M HNO_3_ for regeneration. Both acids effectively restored GO’s adsorption capacity in the first two cycles, maintaining efficiencies above 95%. However, a decline in performance was observed in the third cycle, with HCl-treated GO retaining higher efficiency (92.1%) compared to HNO_3_-treated GO (89.7%). These results suggest that GO can be reused for multiple lithium adsorption cycles with minimal efficiency loss, and that HCl is more suitable for regeneration due to its milder effect on the GO structure. Further regeneration studies are needed using a lower dilution of the regenerating acids.

### 3.8. Adsorption Mechanism

The mechanism of lithium adsorption on GO is primarily governed by electrostatic interactions, ion exchange, surface complexation, and weak π-π interactions, all facilitated by the oxygen-containing functional groups on GO. These functional groups, including hydroxyl, carboxyl, and epoxy groups, play a pivotal role in enhancing lithium adsorption. Experimental observations confirm that the adsorption process is highly pH-dependent, with increased adsorption capacity at higher pH levels, as illustrated in Figure 4. This behavior highlights the dominance of electrostatic interactions, where the negatively charged GO surface at higher pH enhances the attraction between GO and positively charged Li^+^ ions. Ion exchange mechanisms are particularly significant, as evidenced by the decreased competition with H^+^ ions at higher pH. Additionally, FTIR and XPS data reveal the presence of C=O and C–O–C peaks, indicating the possibility of forming stable complexes between Li^+^ ions and oxygen functional groups on GO.

Kinetic modeling highlights lithium adsorption mechanisms, with the PSO model as the best fit, indicating chemisorption driven by electron sharing or exchange between GO and Li^+^ ions. The model indicates that the adsorption rate is directly proportional to the square of the driving force, highlighting the interactions between the GO’s active sites and lithium. Equilibrium isotherm analysis supports these findings, with the Redlich–Peterson (RP) isotherm providing the best fit, indicating a mixed mechanism of monolayer and multilayer adsorption on both homogeneous and heterogeneous sites. The Sips isotherm highlights the role of surface heterogeneity and the functional groups of GO. While weak π-π interactions may stabilize lithium ions, electrostatic and ion exchange mechanisms dominate.

Competition studies confirm GO’s selective adsorption of Li^+^ over Na^+^, dominated by ion exchange. Desorption with acidic solutions (e.g., HNO_3_, HCl) confirms reversibility, highlighting the role of electrostatic and ion exchange mechanisms. The high retention of adsorption capacity across cycles (Figure 8) demonstrates the stability and reusability of GO for lithium recovery. This study links PSO and RP models with mechanistic insights, providing a foundation for optimizing lithium recovery on GO.

## 4. Conclusions

This study demonstrates the potential of graphene oxide (GO) as a highly efficient adsorbent for lithium-ion removal from aqueous solutions. GO was successfully synthesized via the Hummers method and characterized using SEM, FTIR, XRD, XPS, BET, and TGA analyses, confirming its unique structural and chemical properties. Adsorption experiments revealed that GO exhibits a high lithium adsorption capacity of 179 mg/g, with the Redlich–Peterson isotherm providing the best fit for equilibrium data, indicating a combination of multimechanistic adsorption mechanisms. Kinetic studies confirmed that lithium adsorption follows the pseudo-second-order model, suggesting chemisorption as the dominant mechanism. Key parameters, including pH, salinity, and initial lithium concentration, influenced the adsorption performance of GO. Higher pH levels favored lithium uptake due to increased electrostatic interactions, while elevated salinity levels reduced adsorption efficiency due to competitive ion effects. GO also demonstrated strong reusability, maintaining significant adsorption capacity over multiple regeneration cycles, with HCl proving to be the most effective desorbing agent. Compared to existing lithium adsorbents, GO exhibits superior adsorption capacity and selectivity, making it a promising material for lithium recovery from wastewater and brine sources. Future research should focus on scaling up GO-based adsorption systems, optimizing regeneration processes, and evaluating the long-term stability of GO in real-world applications. Overall, this study provides valuable insights into the feasibility of GO for sustainable lithium extraction, contributing to the development of efficient and eco-friendly resource recovery technologies.

## Figures and Tables

**Table 1:** The atomic ratio of the identified elements in the adsorbent.

Name	Peak BE	FWHM eV	Area (P) CPS.eV	Atomic %
C 1s	259.00	4.26	691,735	62.17
O 1s	532.30	3.20	1,045,249	36.98

**Table 2:** Fitting of various kinetic models for the adsorption of Li^+^ onto GO. (V = 100 mL, 20 mg GO, C_0_ = 100 mg/L Li^+^, initial pH 5, 25 °C, and 500 rpm).

Kinetic Model	Parameters	Fitting Quality
Pseudo-First Order	q_e_ = 52.47 mg/g K_1_ = −0.002	R^2^ = 0.871 SSR = 8.71
Pseudo-Second Order	q_e_ = 92.00 mg/g K_2_ = 0.003	R^2^ = 0.997 SSR = 0.001
Elovich	α = 50.00 β = 0.053	R^2^ = 0.922 SSR = 530.17
Intra-Particle Diffusion	K_IP_ = 9.73 C = 26.24	R^2^ = 0.761 SSR = 1621.86
Avrami	q_e_ = 84.64 mg/g K_av_ = 0.211 n_AV_ = 0.942	R^2^ = 0.997 SSR = 22.38

**Table 3:** Fitted isotherm models for lithium adsorption on GO.

Isotherm Model	Isotherm Parameters			SSE	R^2^
	P1	P2	P3		
Langmuir	K_L_ = 1.99	a_L_ = 0.01	q_max_ = 179 mg/g	35.72	1.00
Freundlich	a_F_ = 8.79	b_F_ = 0.50	-	145.9	0.99
Redlich–Peterson	K_R_ = 2.44	a_R_ =0.032	b_R_ = 0.85	20.91	1.00
Sips	K_LF_ = 3.05	a_LF_ = 0.01	n_LF_ = 0.87	22.19	1.00
Tempkin	B = 38.97	a_T_ = 0.11	-	101.2	0.99

**Table 4:** Adsorption isotherm models and adsorption capacities for Li^+^ adsorption using different materials.

Adsorbent	Maximum Capacity (mg/g)	Isotherm Model	Ref.
Lithium/aluminum layered double hydroxides	7.3	Sips	[41]
Granulated H_4_Mn_5_O_12_ (HMO) ion sieve (CTS)	12	-	[12]
Adsorbent derived from spinel lithium manganese oxide	23	-	[42]
Hollow hemispherical mixed matrix lithium adsorbent	25.7	Freundlich	[17]
Spherical layer-structured H_2_TiO_3_ ion sieve	30	-	[11]
Magnetically recyclable Fe-doped Mn oxide spinel	34.8	Langmuir	[14]
Nano-metatitanic acid lithium ion sieve	47	Langmuir	[43]
Iron-doped lithium ion-sieves	53	-	[44]
Modified activated carbon MnO_2_ nanocomposites	89	-	[15]
Date pits impregnated with cellulose nanocrystals and ionic liquid	99	Freundlich	[17]
**Graphene oxide**	**179**	**Redlich–Peterson**	**This study**

## Data Availability

The original contributions presented in this study are included in the article/supplementary material. Further inquiries can be directed to the corresponding authors.

## Supplementary Materials

The following supporting information can be downloaded at https://www.mdpi.com/article/10.3390/ma18143211/s1, Table S1: Isotherm models for single adsorption, Table S2: Kinetic models for single adsorption.

## Abbreviations

The following abbreviations are used in this manuscript:

Li	Lithium
C_0_	Initial lithium adsorbate concentration
Ce	Equilibrium lithium adsorbate concentration
GO	Graphene Oxide
PFO	Pseudo-first order kinetic model
PSO	Pseudo-second order kinetic model
Qe	Capacity at a specific concentration
qmax	Maximum adsorption capacity
Qt	Capacity at time t
R^2^	Coefficient of determination error analysis
SSE	Sum of the square of the errors
