# Supplementary material for: Lithium Adsorption Using Graphene Oxide: Modeling, Regeneration, and Mechanistic Insights
Source: Materials (Basel). 2025 Jul 8;18(14):3211. doi: 10.3390/ma18143211 (PMC12300906; doi:10.3390/ma18143211)
Supplement: Supplementary file 1 [file materials-18-03211-s001.zip › materials-3613430-supplementary.pdf]

*Table S1: Isotherm models for single adsorption*

| Isotherm models | | | |
|---|---|---|---|
| **Model name** | **Equation** | **Description** | **Reference** |
| Langmuir | $q_e = \frac{K_L C_e}{1 + a_L C_e}$ | Considers adsorption as a continuous bombardment of molecules onto a surface with their corresponding desorption or evaporation from the surface with no aggregation at the surface | [1] |
| Freundlich | $q_e = a_F C_e^{b_F}$ | Not limited to monolayer formation and can be applied to formation of multilayers. Adsorption heat does not need to be uniformly distributed on the heterogeneous surface of the isotherm | [2] |
| Redlich-Peterson | $q_e = \frac{K_R C_e}{1 + a_R C_e^{b_R}}$ | Can be applied to heterogenous and homogenous systems as it features both Freundlich and Langmuir models | [3] |
| SIPS | $q_e = \frac{K_{LF} C_e^{n_{LF}}}{1 + a_{LF} C_e^{n_{LF}}}$ | Combines Langmuir and Freundlich isotherm models to predict the heterogeneity of the system- it localizes the adsorption without adsorbate–adsorbate interaction | [4] |
| Temkin | $q_e = B \ln A_T + B \ln C_e$ | Considers the interaction between the adsorbent and the adsorbate by ignoring the extremely large and low concentration values- it assumes that adsorption heat of all molecules in the layer declines linearly rather than logarithmically | [5] |

*Table S2: Kinetic models for single adsorption*

| Kinetic models | | | |
|---|---|---|---|
| **Model name** | **Equation** | **Description** | **Reference** |
| Pseudo-first | $q_t = q_e(1 - e^{-k_1 t})$ | Adsorption is the difference equilibrium adsorption and the adsorbed capacity at time multiplied by the rate constant of the adsorption. The rate of adsorption is proportional to this driving force linearly | [6] |
| Pseudo-second | $q_t = \frac{q_e^2 k_2 t}{1+q_e k_2 t}$ | Adsorption is the difference between the equilibrium adsorption capacity and the adsorbed capacity multiplied by the rate constant. However, in this model, the rate of adsorption is proportional to the square of the driving force indicating each adsorbate occupies two adsorption sites | [7] |
| Elovich | $q_t = \alpha \ln(a\alpha) + \alpha \ln t$ | This model looks into this from a chemisorption kinetics perspective by describing the reduction in rate of adsorption due to increase in surface coverage with time | [8] |
| Avrami's exponential | $q_t = q_e(1 - e^{-k_1 t})^n$ | This model is adapted from Avrami's kinetic decomposition model which is used to evaluate the reaction rate as the fraction of adsorption at time, and the rate constant. It also considers multiple adsorption sites | [9] |
| Weber and Morris Diffusion | $q_t = k_p t^{\frac{1}{2}} + C$ | The equation for the Weber and Morris intraparticle diffusion model is based on some assumptions. Firstly, it assumes that the resistance to mass transfer is only significant at the beginning of the diffusion. Secondly, the concentration governs the radial diffusion process, only constant diffusion occurs in the process | [10] |

## References


[1] I. Langmuir, “A new adsorption isotherm,” *Langmuir, I.*, vol. 40, pp. 1361–1403, 1918.

[2] H. Freundlich, “Colloid and capillary chemistry,” *London:Methuen*, pp. 6–8, 1926.

[3] D. L. Redlich, O. J. D. L., & Peterson, “A useful adsorption isotherm.,” *J. Phys. Chem.*, vol. 63, no. 6, pp. 1024–1024, 1959.

[4] R. Sips, “On the structure of a catalyst surface,” *J. Chem. Phys.*, vol. 16, no. 5, pp. 490–495, 1948.

[5] M. I. Temkin, “Kinetics of heterogeneous catalysis,” *J. Phys. Chem.*, vol. 14, pp. 1153–1158, 1940.

[6] S. Lagergren, “Zur theorie der sogenannten adsorption gelo¨ster stoffe.,” *K. Sven. Vetenskapsakademiens*, vol. 24, no. 4, pp. 1–39, 1898.

[7] G. Ho, Y. S., & McKay, “Pseudo-second order model for sorption processes.,” *Process Biochem.*, vol. 34, no. 5, pp. 451–465, 1999.

[8] W. R. ( Chien, S. H., & Clayton, “Application of Elovich equation to the kinetics of phosphate release and sorption in soils,” *Soil Sci. Soc. Am. J.*, vol. 44, no. 2, pp. 265–268, 1980.

[9] A. R. Lopes, E. C., dos Anjos, F. S., Vieira, E. F., & Cestari, “An alternative Avrami equation to evaluate kinetic parameters of the interaction of Hg (II) with thin chitosan membranes,” *J. Colloid Interface Sci.*, vol. 263, no. 2, pp. 542–547, 2003.

[10] J. C. ( Weber Jr, W. J., & Morris, “Kinetics of adsorption on carbon from solution. J,” *J. Div. Sanit. Eng.*, vol. 89, no. 2, pp. 31–59, 1963.
